# HIV-1 Transmission Patterns in Antiretroviral Therapy-Naïve, HIV-Infected North Americans Based on Phylogenetic Analysis by Population Level and Ultra-Deep DNA Sequencing

**DOI:** 10.1371/journal.pone.0089611

**Published:** 2014-02-26

**Authors:** Lisa L. Ross, Joseph Horton, Samiul Hasan, James R. Brown, Daniel Murphy, Edwin DeJesus, Martin Potter, Anthony LaMarca, Ivan Melendez-Rivera, Douglas Ward, Jonathon Uy, Mark S. Shaefer

**Affiliations:** 1 ViiV HealthCare, Research Triangle Park, North Carolina, United States of America; 2 GlaxoSmithKline, Research Triangle Park, North Carolina, United States of America; 3 GlaxoSmithKline, Stevenage, United Kingdom; 4 Clinique Medicale L’Actuel, Montreal, QC, Canada; 5 Orlando Immunology Center, Orlando, Florida, United States of America; 6 McGill University Health Centre, Montreal, QC, Canada; 7 Therafirst Medical Center, Ft Lauderdale, Florida, United States of America; 8 Centro Ararat, Ponce, Puerto Rico; 9 Dupont Circle Physicians Group, Washington, DC, United States of America; 10 Amicus Therapeutics, Cranbury, New Jersey, United States of America; Centro de Biología Molecular Severo Ochoa (CSIC-UAM), Spain

## Abstract

Factors that contribute to the transmission of human immunodeficiency virus type 1 (HIV-1), especially drug-resistant HIV-1 variants remain a significant public health concern. In-depth phylogenetic analyses of viral sequences obtained in the screening phase from antiretroviral-naïve HIV-infected patients seeking enrollment in EPZ108859, a large open-label study in the USA, Canada and Puerto Rico (ClinicalTrials.gov NCT00440947) were examined for insights into the roles of drug resistance and epidemiological factors that could impact disease dissemination. Viral transmission clusters (VTCs) were initially predicted from a phylogenetic analysis of population level HIV-1 *pol* sequences obtained from 690 antiretroviral-naïve subjects in 2007. Subsequently, the predicted VTCs were tested for robustness by ultra deep sequencing (UDS) using pyrosequencing technology and further phylogenetic analyses. The demographic characteristics of clustered and non-clustered subjects were then compared. From 690 subjects, 69 were assigned to 1 of 30 VTCs, each containing 2 to 5 subjects. Race composition of VTCs were significantly more likely to be white (72% vs. 60%; p = 0.04). VTCs had fewer reverse transcriptase and major PI resistance mutations (9% vs. 24%; p = 0.002) than non-clustered sequences. Both men-who-have-sex-with-men (MSM) (68% vs. 48%; p = 0.001) and Canadians (29% vs. 14%; p = 0.03) were significantly more frequent in VTCs than non-clustered sequences. Of the 515 subjects who initiated antiretroviral therapy, 33 experienced confirmed virologic failure through 144 weeks while only 3/33 were from VTCs. Fewer VTCs subjects (as compared to those with non-clustering virus) had HIV-1 with resistance-associated mutations or experienced virologic failure during the course of the study. Our analysis shows specific geographical and drug resistance trends that correlate well with transmission clusters defined by HIV sequences of similarity. Furthermore, our study demonstrates the utility of molecular and epidemiological analysis of VTCs for identifying population-specific risks associated with HIV-1 transmission and developing effective local healthcare strategies.

## Introduction

With the availability of highly active antiretroviral therapy (HAART) transmission of drug resistant human immunodeficiency virus type 1 (HIV-1) quasi-species has become of increasing concern. While drug resistance acquired through vertical transmission has been well studied, considerably less is known about epidemiology surrounding the transmission of drug resistance in adults. Using phylogenetic analyses of HIV-1 *pol* sequences from HIV-infected subjects, highly similar groups of viruses can be inferred to be clusters of related infections, or viral transmission clusters (VTCs). Previous phylogenetic studies have explored HIV transmission networks among subjects within a relatively confined geographical area [1]–[19] with fewer attempts to determine transmission dynamics in a large multi-site or an international setting [20]–[23]. With the advent of the more sensitive next generation sequencing (NGS) methodologies, ultra deep sequencing (UDS) is being increasingly used to detect low abundance viral variants derived from clinical viral isolates [24].

The viral sequence interrelationships and epidemiological data from antiviral-naïve subjects seeking treatment can provide a framework for better understanding the population dynamics behind drug resistant virus and of HIV-1 transmission networks which may span large geographic regions. In this study we used a phylogenetic approach to identify VTCs in ART-naïve subjects infected with highly related HIV. These subjects, from the continental United States (USA), Canada and Puerto Rico, were seeking HAART treatment through enrollment in the open-label, randomized clinical trial ARIES (Atazanavir-Ritonavir Induction with Epzicom Study; EPZ108859) [25]–[27]. We examined demographic differences between HIV-infected subjects using Sanger and NGS DNA sequencing technologies along with phylogenetic analyses. Responses to therapy for subjects from VTCs were compared to those subjects with non-clustered sequences to better understand the population dynamics behind the spread of HIV infection within study subjects and aid in future design of treatment strategies.

Although our population was restricted to only patients who choose to seek treatment through clinical trial enrollment, it is in agreement with other studies that found the majority of VTCs tend to occur within close geographic proximity and to be associated with men-who-have-sex-with-men (MSM) behaviors. Our study is consistent with reports from other VTC studies that HAART is highly effective in treating this population.

## Materials and Methods

### Ethics Statement

All study subjects provided written informed consent. This study was approved by the ethics review boards used by for the 72 participating centers, which included Ethica Clinical Research Inc., MUHC Biomedical D, Sunnybrook Health (Hth) Sciences Centre (CTR) Research (Res) Ethics Board (B), University (U) Hth Network Res Ethics B, Copernicus Group IRB, Henry Ford Hth System (S) Res Administration, U of South Florida IRB, U and Medical (Med) CTR IRB of East Carolina U, U of Toledo Department (Dept) for Human (HU) Res Protections(P) Biomedical IRB, Whitman-Walker Clinic IRB, Summa Hth S Hospitals (Hp) IRB, Western IRB, U of Kentucky Office (O) of Res Integrity, HU Subjects (SU) Res Committee (MN), Florida Dept of Hth IRB, Kaiser Permanente CTR for Hth Res/Southeast, U of Southern California Hth Sciences Campus IRB, Georgetown University IRB, St. Joseph’s Hp IRB, Chesapeake Research Review, Inc., U of Texas Med Branch O of Res SU P IRB, Loyola U Med CTR IRB for the P of HU SU, Washington U in St. Louis HU Res P O, U of Chicago IRB, U of Southern California School of Medicine IRB, Partners HU Res O, New York Med College Committee for P of HU SU, Thomas Jefferson IRB, Miriam Hp Clinical Res RB, U of Pennsylvania IRB, and the AIDS Res Consortium of Atlanta, Inc. IRB. This study was conducted in accordance with Good Clinical Practice.

### Study Design

Blood plasma-derived HIV samples used in the genotypic analysis were obtained from HIV-infected, ART-naïve subjects during their screening visit to determine eligibility for study enrollment in ARIES. HIV genotyping was performed prospectively on all subjects at screening as an appropriate HIV population genotype obtained by conventional sequencing was a study eligibility criterion. Full descriptions of the study design for ARIES, the subject enrollment criteria and the clinical results from this study have been published previously [26], [27].

A sumary of the clinical trial study primary and secondary outcome measures and their results are also available at http://clinicaltrials.gov/ct2/home (ClinicalTrials.gov Identifier NCT00440947). All pre-therapy samples were obtained during 2007, from a total of 72 centers in the mainland USA, Puerto Rico, and Canada. Subjects were eligible for enrollment if they met all study entry criteria. Primary entry criteria included being: 1) HIV-infected; 2) ART-naïve; 3) a minimum of 18 years of age; 4) plasma HIV RNA ≥1,000 copies/mL with any CD4+ lymphocyte count and, 5) an appropriate HIV genotype. Subjects were ineligible if they had any of the following conditions: 1) HLA-B*5701 allele or hepatitis B surface antigen positive; 2) medical conditions that could compromise their safety or interfere with drug absorption; 3) required use of prohibited medications or; 4) protocol-specified abnormal laboratory values or a creatinine clearance estimated by the Cockcroft-Gault equation of <50 mL/min. All enrolled subjects initially received ritonavir-boosted atazanavir plus abacavir/lamivudine. Subjects with HIV-RNA suppression levels below 50 copies/mL by week 30 were randomized 1∶1 at week 36 to either continue their original regimen or discontinue ritonavir for up to 108 additional weeks of treatment.

### DNA Sequence Analysis

Blood plasma-derived pre-therapy HIV reverse transcriptase (RT) and protease regions of the *pol* gene were analyzed prospectively by population genotyping at the screening visit using TRUGENE (Research Think Tank; GA, USA). The HIV sequence analyzed by TRUGENE region included protease amino acids (AA) 4–99 and reverse transcriptase (RT) AA 38–247. Samples from subjects with confirmed virologic failure or cVF (defined as failure to suppress HIV-1 RNA to <400copies/mL by Week 36 or confirmed virologic rebound to >400copies/mL after suppression to <400copies/mL at any time) during ARIES were analyzed by Phenosense GT at baseline and time of cVF (Monogram Biosciences South San Francisco, California, USA). Nucleoside reverse transcriptase inhibitors (NRTIs), non-nucleoside reverse transcriptase inhibitors (NNRTIs) and protease inhibitor (PI) resistance-associated mutations were defined according to International Antiviral Society USA (IAS-USA) Guidelines from March 2013 (http://www.iasusa.org/sites/default/files/tam/21-1-6.pdf).

HIV RT (reverse transcriptase) and protease for the pre-therapy time-point was also analyzed by UDS as performed by 454 Life Sciences/Roche (Branford, CT, USA) using proprietary primers. The HIV sequences analyzed by UDS included protease AA 10-99 and RT AA 1-251. Median UDS sample read depth over all amino acid positions was 3273 reads (VTC subjects median sample read depth/amino acid position range: 101–15529) and frequency results were censored at ≥1% with a read depth ≥100. Quest Diagnostics (Van Nuys, California, USA) performed all other laboratory tests. HIV RNA concentrations were measured by the Roche Cobas Amplicor HIV-1 Monitor or Ultrasensitive Monitor Test (Roche Diagnostic Systems, Branchburg, NJ). Clade analysis was performed using RIP 3.0 (http://www.hiv.lanl.gov/content/sequence/RIP/RIP.html). RIP was configured to perform query alignments against the HIV database standard HIV-1 subtype consensus alignments. Each *pol* sequence was analyzed with a sliding window of decreasing sizes 200,150, and 100 nucleotides (nt) until a subtype could be determined or was marked as unknown.

### Phylogenetic Reconstruction and VTC Prediction

HIV-1 RT and protease nucleotide sequences obtained by population genotype sequencing from ART-naïve subjects at the screening visit were concatenated and subsequently aligned using the software MUSCLE [28]. The resulting alignment was used to reconstruct a neighbor joining (NJ) tree using the programs DNADIST (default F84 matrix) and NEIGHBOR from the PHYLIP 3.67 package [29]. In estimating genetic distances, the F84 model incorporates different rates of transition and transversion and also allows for different frequencies of the four nucleotides. HIV-1 Subtype K sequence AJ24935 was used as an outgroup for the tree and 1000 bootstrap replicates were used to estimate node support. A VTC was defined as any operating taxonomic unit (OTU) cluster having 2 or more sequences with a median confidence estimate of ≥95% of 1000 bootstrap replicates using a custom Perl script. The choice of classifying OTUs based on the median rather than an absolute value threshold allowed some leniency for comparing VTC predictions across datasets.

HIV samples from ARIES participants identified by population sequencing as being in VTCs were sent for further HIV RT and protease UDS analysis along with pre-therapy samples from all ARIES cVF participants and a random subset selected from all ARIES participants with remaining pre-therapy HIV samples. Sequence concatenation, alignment and phylogenetic analyses were as described above for all samples. A single consensus sequence was generated for each subject in this dataset using the majority nucleotide sequence at each codon. Phylogenetic trees were visualized and drawn using Archaeopteryx v0.9813 tree viewer [30].

We combined the two resultant phylogenetic analyses to further filter our final selection of VTCs by stipulating that any VTC must have at least two shared participants predicted by both population and UDS datasets. The minimum shared participants were considered to be high confidence VTC predictions and were analyzed further for demographic trends.

Descriptive statistics were primarily used in presenting the data, although Mann–Whitney U test (2-tailed) was used for comparing population medians. Data comparisons between subjects with clustered versus those with non-clustered virus were carried out using the Fisher’s exact test (2-tailed).

## Results

HIV RT and protease population genotyping was prospectively attempted using plasma samples collected from 726 subjects who were initially screened for the study. HIV population genotype was obtained from a total of 690 unique ART-naïve subjects from sites located in the continental United States, Puerto Rico and Canada and an appropriate genotype was a requirement for later study enrollment. These 690 subjects were from 72 sites; each site recruited a median of 8 subjects (range 1–52 subjects). For the 36 samples for which there were no initial population genotype data, typically no population genotype was obtained post analysis although for a small subset of subjects two genotypes were obtained. The most recent genotype was used if a subject had been allowed to rescreen for the study after failing to meet other entry criteria and was assigned a new subject identification. UDS analysis was performed after the initial phylogenetic analysis of the population sequence on a subset of 350 samples, which included baseline or screen samples from all subjects identified as being in a VTC by population genotype phylogenetic analysis, all subjects with cVF and a randomly selected subset of 250 study subjects. UDS results were obtained for 325 baseline or screen samples. No results were obtained for some samples due to technical limitations of the assay (typically insufficient plasma or HIV-1 RNA <2000 copies/mL).

Most subjects (664/690; 96%) were infected with subtype B virus, with further subjects detected with viral subtypes A (n = 6), C (n = 11), D (n = 1), G (n = 7) or indeterminate (n = 1).

### Comparisons of VTCs Predictions from Two Datasets

A total of 40 VTCs, comprising virus from 91 subjects, were predicted by NJ tree reconstruction using the population sequencing dataset (Table S1). In comparison, NJ analysis of the UDS dataset, yielded 31 VTC predictions representing viruses from 71 subjects. A total of 15 viral genotypes from subjects who were part of VTCs in the NJ population sequence tree were not identified by UDS analysis as being in these same clusters. Conversely, a total of 15 genotypes from subjects who were part of VTCs in the UDS phylogenetic analysis were not similarly clustered by population sequencing.

**Figure 1:**
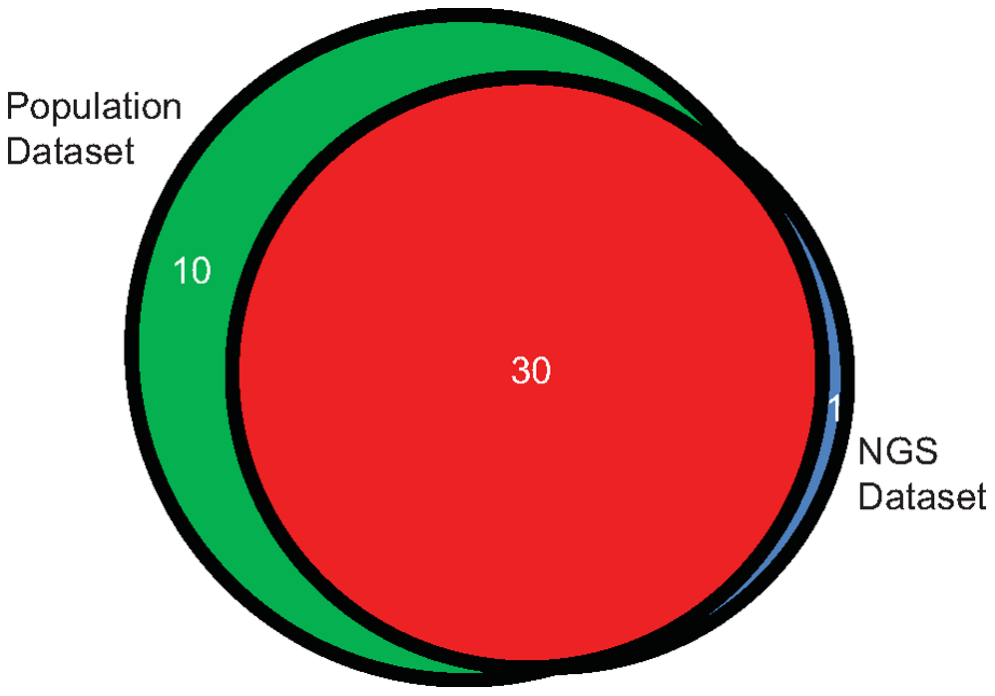
Prediction of VTCs by two studies using HIV-1 sequences obtained from pre-therapy subjects. Shown are comparisons of VTC numbers as predicted by NJ tree-reconstruction of either population-based, ultra-deep sequencing (UDS) or both datasets. See Methods and Materials for VTC prediction methodology.

**Figure 2:**
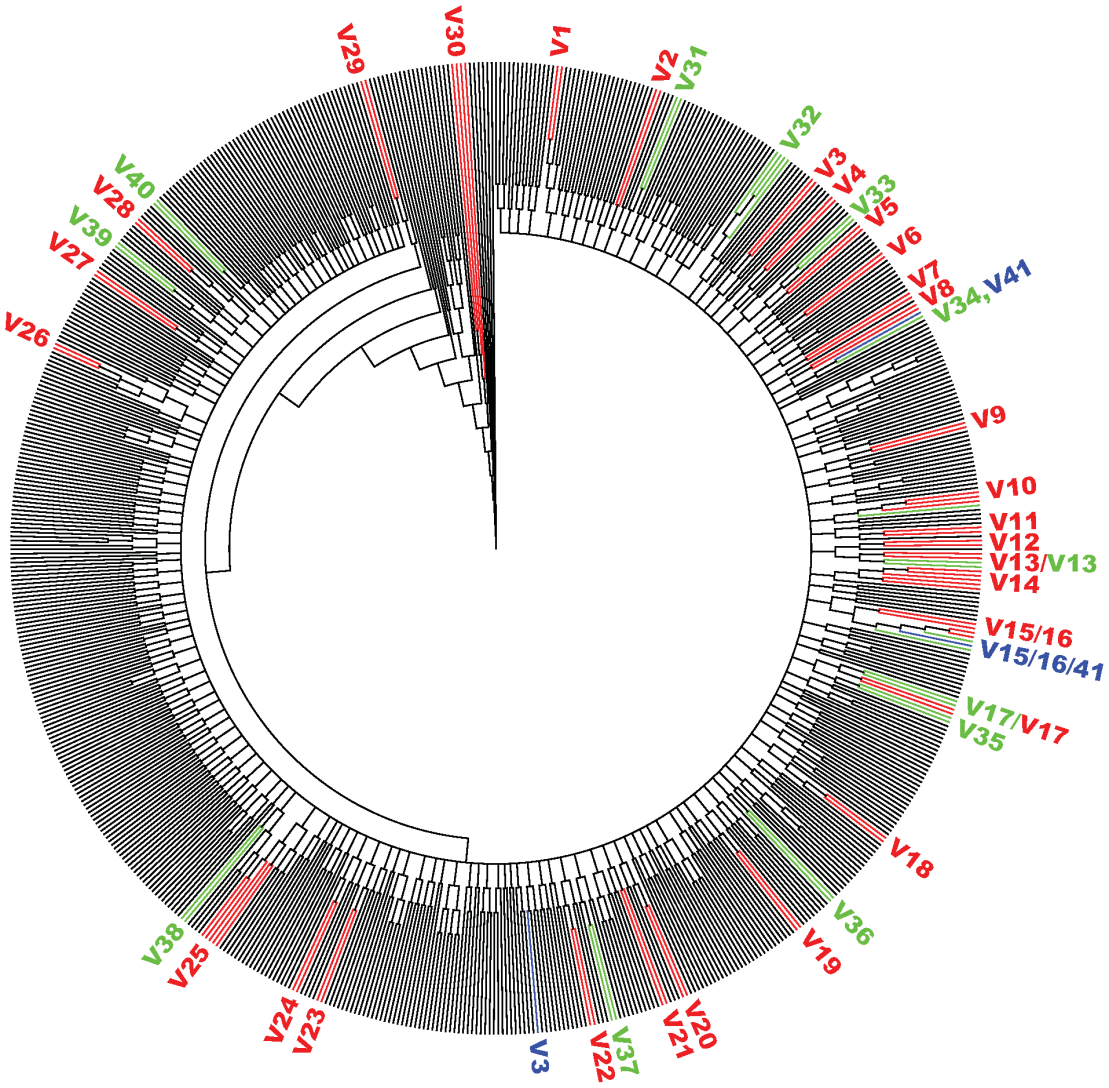
Neighbor-joining (NJ) tree based on population-genotyped HIV sequences from 690 pre-therapy subjects. High-confidence VTCs are labeled in red (1–30). These VTCs all represent 2 or more clustered subjects with a minimum confidence level of 95% in the (1000 bootstrap NJ replicates) for both population and UDS datasets. VTCs predicted only by the population or UDS data are shown in green and blue, respectively. VTCs where sequences are subsets of population or UDP VTCs have multiple colored labels. A more detailed phylogenetic tree with the OTUs labeled appears in Figure S1.

The majority of the predicted VTCs by either method overlapped; however there were 10 and 1 VTCs predicted by the population and UDS analyses, respectively, which were not identified by the other method. In total, 30 VTCs (composed of 69 subjects) were predicted by both methods and considered here as high confidence VTC predictions or VTCs (Figure 1). Composite NJ trees using both datasets are shown in Figure 2 and Figure S1.

### Comparison between the Non-clustered Subject and VTC Subject Populations

The epidemiological and pre-treatment clinical characteristics for the overall cohort population, VTCs and non-clustered subjects are given in Table 1. The subjects within VTCs were demographically similar in many respects to the non-clustered population. However, they were significantly more likely to have reported MSM as a transmission risk (p = 0.001) and be from Canadian study centers (p = 0.003). VTC were significantly more likely to be white (72% vs. 60%; p = 0.04) and non-VTCs were significantly more likely to be black (22% vs. 34%; p = 0.04). There were no VTCs where the subjects did not list any risk factor for acquiring HIV.

**Table 1:** Comparison of population demographics across study cohort and viral transmission clusters (VTC).

ARIES Cohort	Total cohort (n = 690)	VTC Subjects (n = 69)	Non-clustered Subjects (n = 621)	p-value
Median Age (years)	38	36	38	0.29^a^
Male Gender	582 (84%)	64 (93%)	518 (83%)	0.05^b^
Median HIV-RNA (log_10_copies/mL)	5.05	5.05	5.05	0.90^a^
HIV-RNA ≤100,000 copies/mL	322	30	292	0.61^b^
Median CD4 (cells/mm^3^)	203.5	215	203	0.29^a^
CDC Classification	n = 516	n = 60	n = 456	
CDC Class A	352 (68%)	45 (75%)	307 (67%)	0.30^b^
CDC Class B	98 (19%)	8 (13%)	90 (20%)	0.29^b^
CDC Class C	66 (13%)	7 (12%)	59 (13%)	1.00^b^
Race	n = 689	n = 69	n = 620	–
White	419 (61%)	50 (72%)	369 (60%)	**0.04** b
Black/African descent	226 (33%)	15 (22%)	211 (34%)	**0.04** b
Asian	14 (2%)	2 (3%)	12 (2%)	0.64^b^
Other	44 (6%)	4 (6%)	40 (6%)	1.00^b^
Risk Factors				
MSM	344 (50%)	47 (68%)	297 (48%)	**0.001** b
Heterosexual	156 (23%)	15 (22%)	141 (23%)	1.00^b^
IV Drug Use	23 (3%)	0 (0%)	23 (4%)	0.15^b^
Transfusion	8 (1%)	0 (0%)	8 (1%)	1.0^b^
Occupational	3 (0%)	0 (0%)	3 (0%)	1.0^b^
Other	17 (2%)	0 (0%)	17 (3%)	0.4^b^
Hepatitis B	13 (2%)	0 (0%)	13 (2%)	0.63^b^
Hepatitis C+ (n = 689, 69, 620)	54 (8%)	2 (3%)	52 (8%)	0.15^b^
Geographic location				
Mainland USA Subjects	542 (79%)	46 (67%)	496 (80%)	**0.02** b
Puerto Rican Subjects	41 (6%)	3 (4%)	38 (6%)	0.79^b^
Canadian Subjects	107 (16%)	20 (29%)	87 (14%)	**0.003** b

a Mann–Whitney U test;

b Fisher’s exact test.

The specific “n” is noted when demographic data was obtained for a smaller number of subjects. Significant findings (p<0.05) are highlighted in bold.

### Composition of Subjects in High Confidence VTCs

Subject compositions within each of the 30 high confidence VTCs are given in Table 2. Almost all of the VTCs (97%) included men while only 5 female participants were found across 4 VTCs. Of these 4 VTCs, one contained only women (one African-descent USA subject and one African-descent Canadian subject) and 2 contained one female and one male (all white USA participants), while the remaining female-containing VTC was composed of 4 participants (one African-descent female and 3 white males; all from different USA cities). Six VTCs were multiracial in composition, while 18 VTCs included only white participants and 6 VTCs were exclusively of African descent.

**Table 2:** Viral transmission clusters or VTC attributes and demographics.

Cluster Attributes	# of VTCs	Total # of subjects within these clusters
>2 subjects	4	17
Includes males	29	67
Includes females	4	10
Female only	1	2
White (White/Caucasian/European Heritage) only	18	40
Black (African Heritage) only	6	12
≥2 races within cluster	6	17
≥1 Hispanic subject	8	19
≥1 subject MSM Risk Factor	24	57
Includes both heterosexual and MSM Risk Factor	5	15
≥1 subject with IVDU	0	0
Heterosexual only Risk Factor	4	8
All subjects in the VTC treated in the same state/province	19	38
All subjects in the VTC treated in the same city	17	34
≥2 subjects treated in the same city	20	47
≥2 subjects treated in the same state/province	22	51
Clusters that included subjects from mainland USA and PuertoRico or subjects from mainland USA and Canada	3	9
VTC that include ≥1 Canadian subjects^a^	8	23^a^
VTC that include ≥1 Puerto Rican subjects^b^	2	4^b^
HCV co-infected	2	7
HBV co-infected	0	0
≥1 subject CDC Class C	7	19
≥1 subject HIV-RNA >100,000 copies/mL	24	57
≥1 subject CD4>350 cells/mm^3^	11	27
≥1 subject CD4<250 cells/mm^3^	19	47

a Not all subjects within each cluster were Canadian.

b Not all subjects within each cluster were Puerto Rican.

Women within the VTCs reported only heterosexual HIV infection risks. Risk factors for the 64 men in VTCs included MSM (70%) and heterosexual contact (13%) or both (3%) MSM and heterosexual contact risk factors. Four VTCs were composed of participants with only heterosexual contact listed as an HIV transmission risk while 5 VTCs, including one of the largest-sized 5 member clusters, had participants who noted either heterosexual and/or MSM contact as transmission risks. The majority of VTCs (80%) contained at least one subject with MSM risk factor.

The majority of VTCs were composed of subjects all receiving antiretroviral therapy (ART) treatment in the same city (57%) or state/province (63%). However, 3 VTCs contained subjects from both Canada and the USA (including Puerto Rico). The majority of the VTCs (29/30) identified by both DNA sequencing analyses were infected with subtype B HIV-1 while the remaining VTC was comprised of 3 white males and one African Heritage female; all from the USA who were infected with subtype A HIV-1.

The majority (87%) of the 30 high confidence VTCs were comprised of 2 subjects while 4/30 VTCs were composed of >2 subjects including 2 VTCs with 5 subjects, and VTCs with 3 and 4 subjects. The comparison of these VTCs containing >2 subjects is summarized in Table 3.

**Table 3:** Comparison of viral transmission clusters (VTCs) with more than two subjects.

VTC(n)	Ethnicity(Count)^a^	Geographic Location(n)^b^	Risk Factors (n)	CD4 cells/mm^3^(Min-Max)	Viral Load log_10_copies/mL (Min-Max)
3	White (3)	Canadian City/Province A (2),Canadian city/province B (1)	MSM(3)	132–318	3.0–5.7
4	African-Heritage (Female),White (3)	All Different USA Cities/States	Hetero (Female),MSM (1), None (2)	19–507	3.3–6.1
5	White (5)	Canada City/Province (3),US city/state X (2)	MSM(5)	153–282	3.6–5.5
5	White (3), African-Heritage (1),Asian-Heritage (1)	Canadian City/Province A (2),Canadian city/province B (3)	MSM (3), Hetero (1),None (1)	19–401	4.3–5.8

a All males unless otherwise annotated.

b Anonymized locations: A,B,X,.

All subjects in these larger VTCs were males, except for a single female in a 4 member VTC. Most males reported MSM or no risk factors while the female subject reported only heterosexual risk factors. In 3 of these larger-sized VTCs at least 2 of the members were from the same Canadian city and province; one of these VTCs also included 2 subjects from the same USA city and state. The remaining VTC had a more complex geographic distribution with all members in different locations across the USA. All subjects in VTCs with more than 2 members were Hepatitis B/Hepatitis C negative and had similar ranges of CD4 and viral loads.

### Comparisons of HIV-1 Population Genotype Resistance Mutations

By population genotyping, IAS-USA defined RT (including minor NNRTI mutations) and major PI HIV resistance-associated mutations were detected in virus from 151/621 (24%) of the non-clustered subjects, and in significantly fewer (p = 0.002) VTC subjects (6/69; 9%) as summarized in Table 4. Minor PI mutations were excluded from analysis since 96% of subjects had virus with one or more minor PI mutations. When minor NNRTI and NRTI mutations were excluded, major NRTI, NNRTI and PI mutations were detected in virus from 110 (18%) of the non-clustered subjects and from 6 (9%) of VTC subjects.

**Table 4:** Summary of HIV resistance mutations obtained from ultra-deep sequencing (UDS) study.

ARIES Cohort	Total Subjects(n = 690)	Subjects in VTCs(n = 69)	Non VTC Subjects(n = 621)	p-value
**HIV Population Genotype Resistance Mutations**				
Any NRTI, NNRTI or major PI mutation	157 (23%)	6 (9%)1A	151 (24%)1B	**0.002**
Major NRTI, NNRTI, PI mutation	116 (17%)	6 (9%)2A	110 (18%)2B	0.06
Any NRTI mutation	29 (4%)	0 (0%)	29 (5%)3B	0.10
Any NNRTI mutation	126 (18%)	4 (6%)4A	122 (20%)4B	**0.003**
Any major NNRTI mutation	84 (12%)	4 (6%)5A	80 (13%)5B	0.12
Any major PI mutation	24 (3%)	2 (3%)6A	22 (4%)6B	1.00
**UDS HIV Genotype Resistance Mutations (n = 69)**				
Any NRTI, NNRTI or major PI mutation	NA	20 (29%)1C	NA	NA
Major NRTI, NNRTI, PI mutation	NA	15 (22%)2C	NA	NA
Any NRTI mutation	NA	0 (0%)	NA	NA
Any NNRTI mutation	NA	13 (19%)4C	NA	NA
Any major NNRTI mutation	NA	8 (12%)5C	NA	NA
Any major PI mutation	NA	7 (10%)6C	NA	NA

a Analyzed for HIV from VTC subjects only.

1A **RT:** K103N, E138A, Y181C, H221Y; **PR:** M46I, Q58E.

1B **RT:** M41L, D67N, K70R, V75I, V90I, A98G, K101E, K101P, K103N, K103S, V106I, V106M, V108I, E138A, E138G, E138K, Q151M, V179D, Y181C, M184I, M184V, Y188L, G190A, L210W, T215Y, K219E, K219Q, H221Y, P225H, M230I; **PR:** V32I, M46I, M46L, I54M, Q58E, V82A, N83D, I84V, L90M.

1C **RT:** K101E, K103N, V106I, E138A, E138G, E138K, Y181C; **PR:** D30N, M46I, M46L, Q58E, V82A.

2A **RT:** K103N, E138A, Y181C, H221Y; **PR:** M46I, Q58E.

2B **RT:** M41L, D67N, K70R, V75I, K101E, K101P, K103N, K103S, V106M, V108I, E138A, E138K, E138G, Q151M, Y181C, M184I, M184V, Y188L, G190A, L210W, T215Y, K219E, K219Q, H221Y, P225H, M230I; **PR:** V32I, M46I, M46L, I54M, Q58E, V82A, N83D, I84V, L90M.

2C **RT:** K101E, K103N, E138A, E138G, E138K, Y181C; **PR:** D30N, M46I, M46L, Q58E, V82A.

3B **RT:** M41L, D67N, K70R, V75I, Q151M, M184I, M184V, L210W, T215Y, K219E, K219Q; **PR:** –.

4A **RT:** K103N, E138A, Y181C, H221Y; **PR:** –.

4B **RT:** V90I, A98G, K101E, K101P, K103N, K103S, V106I, V106M, V108I, E138A, E138K, E138G, V179D, Y181C, Y188L, G190A, H221Y, P225H, M230I; **PR:** –.

4C **RT:** K101E, K103N, V106I, E138A, E138K, E138G, Y181C; **PR:** –.

5A **RT:** K103N, E138A, Y181C, H221Y; **PR:** –.

5B **RT:** K101E, K101P, K103N, K103S, V106M, V108I, E138A, E138G, E138K, Y181C, Y188L, G190A, H221Y, P225H, M230I; **PR:** –.

5C **RT:** K101E, K103N, E138A, E138G, E138K, Y181C; **PR:** –.

6A **RT:** K103N, E138A, Y181C, H221Y; **PR:** M46I, Q58E.

6B **RT:** –; **PR:** V32I, M46I, M46L, I54M, Q58E, V82A, N83D, I84V, L90M.

6C **RT:** –; **PR:** D30N, M46I, M46L, Q58E, V82A.

Fisher’s Exact test was used to determine if there was a significant difference in the occurrence of resistance mutations between clustered and non-clustered subjects. Significant findings (p<0.05) are highlighted in bold.

NNRTI mutations were the most commonly detected resistance-associated mutations and the differences between VTC and non-clustered groups was significant (p = 0.003). Four (6%) VTC subjects had virus with NNRTI mutations that included the variants K103N, E138A, Y181C and H221Y. In contrast, significantly more non-clustered subjects had virus with NNRTI mutations (122/621 or 20% of viral samples). The mutations observed included all of the previously listed variants as well as V90I, A98G, K101E, K101P, K103S, V106I, V106M, V108I, E138G, E138K, V179D, Y188L, G190A, P225H and M230I.

No virus from VTC subjects had NRTI mutations, compared with 29/621 (5%) of the non-clustered population. The NRTI viral mutations observed in non-clustered subjects included M41L, D67N, K70R, V75I, Q151M, M184I/V, L210W, T215Y, and K219E/Q. There were two major PI mutation (M46I, Q53E) detected in two (3%) viruses from VTC subjects as compared with the subjects with non-clustered virus where 22/621 (4%) contained major PI mutations. In this latter group, most of the subjects (18/22) had virus with a single major PI mutation in addition to minor variants. Within VTCs where major mutations were detected, no other subjects within the VTC had major mutations. However, minor PI mutation profiles of viruses within a VTC were relatively consistent (Table S1).

### Resistance Mutations Detected by UDS Analysis

The HIV RT and protease UDS genotypic results obtained for the 69 VTC subjects were analyzed for the presence of IAS-USA-defined NNRTI, NRTI, and major PI resistance mutations (Table 4). UDS analysis detected NRTI, NNRTI or major PI resistance mutations in virus from 20/69 (29%) of VTC subjects (from 13 VTCs) compared to virus from 6/69 (9%) of VTC subjects detected by population sequencing (from 6 VTCs). Most mutations detected by UDS were at frequencies of <6%. When UDS results were filtered to include only mutations detected at a frequency of ≥6%, then 5 of the 6 VTC subjects identified by population genotyping as having virus with IAS-USA-defined NNRTI, NRTI, and major PI resistance mutations were similarly identified using UDS, suggesting that the difference between UDS and PG was primarily due to newly detected mutations with <6% frequency. The remaining (6^th^) virus was identified by population sequencing as containing the major mutation H221Y but this mutation was not observed by UDS at a frequency above the 1% cutoff.

There were 18 additional NNRTI, NRTI, and major PI resistance mutation observed with UDS that were detected with a prevalence of between ≥1 and <6% of which 12/18 (67%) were different types of NNRTI mutations (major: K101E, K103N, E138A, E138G, E138K; minor: K101E, V106I, E138A, E138G, E138K). The remaining 6/18 types of mutations detected at low prevalence were major PI mutations (D30N, M46I, M46L, V82A). The newly detected mutations that were observed in >1 viral genotype were the major PI mutations M46I (2 subjects) and V82A (2 subjects), and the RT mutation V106I (7 subjects).

A total of 16 subjects had virus with NNRTI and major PI mutations detected by UDS that were not detected by population sequencing with the frequencies of <6%. For the major PI mutations, these were detected by UDS in virus from 6 subjects. These PI major mutations included D30N, M46I, M46L and V82A with the D30N and M46I mutations occurring in 2 separate samples from the same VTC. For 10 viral samples from 8 VTCs, NNRTI mutations were detected only by UDS and at frequencies <6% and included K101E, K103N, V106I, E138A, E138G, and E138K.

### Results on Therapy

Of the 690 screened subjects, a total of 515 subjects met all enrollment criteria and initiated antiviral therapy within the ARIES clinical study. Most (60/69; 87%) of the screened subjects in the VTCs were eligible and elected to enroll in the study. Treatment during the first 36 weeks included two nucleoside reverse transcriptase inhibitors (NRTIs; abacavir/lamivudine) and a ritonavir-boosted PI (atazanavir). Virologically suppressed subjects were randomized at Week 36 to discontinue the ritonavir from their ART regimen or to continue the original treatment regimen. Of the 515 enrolled subjects, 33 experienced cVF (defined as failure to suppress HIV-RNA to <400 copies/mL by Week 36 or confirmed viral rebound at any time through 144 weeks on therapy from <400 copies/mL to ≥400 copies/mL). The majority (30/33) of cVF subjects had non-clustered viral sequences. Fourteen of these subjects met cVF by week 36, eight subjects post week 36 through Week 84, and eight additional subjects post week 84 through week 144.

In contrast, the 3 VTC subjects all met cVF relatively late in the study, after week 84, and were in different VTCs, including two in two-member VTCs and one from the five member VTC. The 3 subjects with cVF were male, as were all of the subjects within their VTCs (Table 5). All three subjects with cVF were negative for Hepatitis B and C. Within their respective VTCs, each had the highest viral load (VL) and the lowest CD4 in two of the VTCs. Two of these subjects with cVF reported MSM as a risk factor and the remaining subject had heterosexual risk. At the start of the study, by population sequencing, only one of these subjects who later met cVF had virus with a major NNRTI resistance mutation (Y181C) but all three had virus with minor PI mutations. UDS analysis confirmed all cVF population sequencing mutations and also detected minor PI resistance-associated mutations in virus at low prevalence (<6%) in one subject. Within these 3 VTCs, one subject from a 2 member cluster failed to meet all study eligibility requirements and was not enrolled, but all of the non-cVF subjects who were in the remaining 2 VTCs initiated drug treatment and maintained sustained viral suppressed with HIV-RNA <50 log_10_ copies/mL at Week 144.

**Table 5:** Demographics for confirmed virologic failure (cVF) containing VTCs.

VTC(n)	Race/Ethnicity(Count)	GeographicLocation (n)	Risk Factors (n)	CD4 cells/mm^3^(Min-Max)	Viral Loadlog_10_ copies/mL(Min-Max)	PG Resistancemutations	Additional UDSResistancemutations
2	White(1), MixedRace/Hispanic(cVF)	Same USA statebut different cities	Heterosexual(cVF), None (1)	185 (cVF) - 731	4.6–4.8 (cVF)	PI minor: V77I, I93L (cVF);PI minor: V77I, I93L	None
2	White(2)	Same USA city	MSM (cVF), MSM/Hetrosexual(1)	49 (cVF) - 234	5.0–5.9 (cVF)	Major NNRTI: Y181C, PI Minor:L10I,I62V,V77I,I93L (cVF); PIMinor: L10I,I62V,L63P,V77I,I93L	None (cVF); PI minor:M36I(6%), I64L(10%),PI Major: V82I(1%)
5^a^	White(3+cVF),White/Hispanic(1)	Canada City/Province B (3),USA city/state (1+cVF)	MSM(5)	153–282;215(cVF)	3.6 - 5.5(cVF)	PI Minor^b^: I62V,L63P,A71T,I93L;PI Minor: M36I,I62V,I93L (cVF);PI Minor: L63P,V77I,I93L; PIMinor: L63P,V77I,I93L; V77I,I93L	

a Non-cVF subject Hepatitis C positive.

b PI Minor: G16E(2%), M36I(2%), I64L(1%); PI Minor: L10I(2%), L63P(3%), V82I(1%) (cVF); NNRTI Minor: V106I(4%); NNRTI Minor: V106I(1%), PI Minor: A71T(1%), A71V(1%), 82(13%); PI Major: M46I(2%), PI Minor: L10I(1%), L63P(1%).

## Discussion

Phylogenetic analysis of HIV using population sequencing and UDS data identified 40 and 31 VTCs, respectively, from 690 antiviral-naïve HIV-infected subjects residing in Puerto Rico, Canada and mainland USA. A total of 30 VTCs comprising 69 subjects were commonly identified by both DNA sequencing methods. Discordances in the number and content of VTCs are likely due to minor differences in the HIV regions sequenced by two approaches as well the number of subjects. Furthermore, population sequencing data could contain nucleotide mixtures while the consensus UDS sequence is created from the majority nucleotide codon sequence.

The majority of subjects in both the non-clustered population and VTCs were male and white. There were significantly more subjects within the VTCs who reported MSM risk factors or from sites in Canada. While many VTC subjects were receiving treatment in the same city or state/province, 37% of the VTCs included subjects who resided in different geographical locations, including cluster composed of subjects from both the mainland USA and Puerto Rico or from both USA and Canada. Our study does not have an extensive longitudinal pre-therapy collection period (all samples were collected in 2007) so the finding that 10% (69/690) of the screening population were part of VTCs and that these networks sometimes encompassed wide geographic areas was unexpected. Recently published findings from a large Center for Aids Research (CFAR) network phylogenetic analysis at five U.S. sites which included both ART-naïve and ART-experienced patients had a higher proportion (24%) of VTCs, with the majority of VTCs (89%) confined to a single site, with <11% of VTCs encompassing two sites and only one VTC associated across three sites [22]. Clustering was associated with the lack of ART use (p<0.001) as well as being marginally associated with MSM/IDU risk behaviors (p = 0.06). Viral phylogenetic analysis of newly diagnosed HIV-infected patients from 25 European countries and Israel European participating in the SPREAD project found that 31.2% were part of a VTC, with the majority of these (74.2%) from within a single European country, while 15.6% clustered with individuals from countries without a common border [23]. Clustering was also significantly associated with MSM behavior (p<0.0001) and subtype B viral infection (p<0.0001).

A possible consequence of rapid HIV transmission through VTCs could have been a local increase in the transmission of drug resistant strains, as sexual transmission of HIV has been reported to select for highly fit drug-resistant and persistent variants [31]. In the European/Israeli SPREAD surveillance project, transmitted drug resistance was significantly more prevalent (p = 0.03) in VTC than non-clustered subjects. However, this was not observed in our study or in the USA Centers for AIDS Research (CFAR) surveillance study [22] where patients in VTCs were less likely to have resistance mutations (p<0.001) than patients with non-clustered sequences. There is a much higher proportion of Subtype B virus in the latter two studies (>96%) than in the SPREAD study (66%). The SPREAD study also found numerous differences between patients infected with subtype B virus compared to non-subtype B with subtype B subjects more often MSM and recently infected (both P<0.001) [23]. Drug resistance was detected in virus from the non-clustered population at a higher prevalence (24%) than in the VTCs (6%). In particular, major PI mutations were rarely observed and no NRTI mutations occurred within the VTCs which is consistent with data from other transmission networks [4], [14]. One possible explanation could be the reduced fitness of virus bearing major PI mutations as compared with viruses bearing NNRTI mutations which can persist *in vivo* for years in the absence of direct drug pressure [32], [33].

The relatively lower incidence of transmitted drug resistance within the VTC population as compared to the incidence of transmitted drug resistance detected in the non-clustered population could be attributed to the spread of the virus from source partners who are unaware of their infection status. This would be consistent with other reports of onward transmission in the MSM population in Brighton, UK and Montreal, Canada which have suggested that most of the new infections in these communities appear to arise from individuals whose infection was undiagnosed at the time of transmission or those recently infected with a higher viral load [32], [33]. In our study, HIV-1 infected Canadian subjects were significantly more likely to be part of a VTC than HIV-infected subjects from either the continental U.S. or Puerto Rico. This could be due to better intervention strategies on the part of Canadian healthcare authorities to encourage HIV testing for at risk individuals as well as increased emphasis on early treatment for HIV-infected subjects. A recent analysis of primary or early infection in HIV-infected MSM subjects from Montreal observed an ongoing increase in the detection of larger clusters containing more than 5 members which was attributed to a disproportionate contribution of subjects in the primary or early stage infection in outward transmission [2]. This would suggest that intervention strategies may not have been uniformly successful. Data from an earlier study on HIV transmission in MSM living in Montreal indicated that 57.8% of study subjects who engaged in non-couple sex (n = 965) were unaware of their partner’s HIV status at last sexual encounter. For MSM subjects who engaged in single night only encounters (n = 465) that percentage increased to 79.1% [34]. Another earlier study in HIV-1 serodiscordant couples in Africa demonstrated that transmission rates in persons diagnosed with AIDS are increased due to high plasma HIV-1 RNA during this period of time, which underscores the importance of early detection and treatment in persons infected with HIV [35].

The VTC demographic and genotyping results from our study are in line with models which suggest that HIV can be spread by HIV-infected persons who may be unaware of their infection status. Limitations of our analysis are the lack of behavioral or duration of infection data which could have provided additional insights. Additionally, there could be potential subject selection bias since our study was restricted to patients who had elected to seek HIV treatment through clinical trial enrollment.

Phylogenetic analyses of population sequence and UDS data produced similar results for VTC predictions. The respective VTC cut-off values of 95% bootstrap NJ tree replicates is highly conservative. Relaxing those constraints could broaden the size and number of VTCs. Nonetheless, we purposely selected more cautious and robust criteria for supporting VTC assignments.

Data from our analysis is in agreement with other studies that have found that although a small number of transmission clusters can span wide geographic areas, the majority of VTCs tend to occur within close geographic proximity and VTCs are associated with MSM behaviors. Study subjects in VTCs had a lower incidence of transmitted drug resistance than subjects with non-clustered virus (9% vs. 24%; p = 0.002) and those who elected to initiate ART were also less likely to experience cVF than subjects with non-clustered virus (5% (3/60) subjects vs. 7% (30/455)). These results underscore the importance of HAART in early chronic infection and the need to further develop local healthcare strategies that encourage HIV testing and behaviors that reduce transmission risk.

## Supporting Information

Figure S1 Neighbor-joining (NJ) tree based on population-genotyped HIV sequences from 690 pre-therapy subjects as shown in Figure 1 but with sequence OTUs labeled. High-confidence VTCs are labeled in red (1–30). These VTCs all represent 2 or more clustered subjects with a minimum confidence level of 95% in the (1000 bootstrap NJ replicates) for both population and UDS datasets. VTCs predicted only by the population or UDS data are shown in green and blue, respectively. VTCs where sequences are subsets of population or UDP VTCs have multiple colored labels.
(TIF)

Table S1 Composition and support for viral transmission clusters (VTCs) using neighbor joining tree analysis of two sequencing datasets.
(XLSX)
